# Higher field reduced FOV diffusion-weighted imaging for abdominal imaging at 5.0 Tesla: image quality evaluation compared with 3.0 Tesla

**DOI:** 10.1186/s13244-023-01513-7

**Published:** 2023-10-15

**Authors:** Yunfei Zhang, Ruofan Sheng, Chun Yang, Yongming Dai, Mengsu Zeng

**Affiliations:** 1 Shanghai Institute of Medical Imaging, Fudan University, Shanghai, 200032 China; 2 Central Research Institute, United Imaging Healthcare, Shanghai, 201800 China; 3 Department of Radiology, Zhongshan Hospital, Fudan University, Shanghai, 200032 China; 4 School of Biomedical Engineering, ShanghaiTech Univerisity, Shanghai, 200032 China

**Keywords:** Diffusion magnetic resonance imaging, 5.0 Tesla, Ultra-high-field MRI, Image quality, rFOV-DWI

## Abstract

**Objective:**

To evaluate the image quality of reduced field-of-view (rFOV) DWI for abdominal imaging at 5.0 Tesla (T) compared with 3.0 T.

**Methods:**

Fifteen volunteers were included into this prospective study. All the subjects underwent the 3.0 T and 5.0 T MR examinations (time interval: 2 ± 1.9 days). Free-breathing (FB), respiratory-triggered (RT), and navigator-triggered (NT) spin-echo echo-planner imaging-based rFOV-DWI examinations were conducted at 3.0 T and 5.0 T (FB_3.0 T_, NT_3.0 T_, RT_3.0 T_, FB_5.0 T_, NT_5.0 T_, and RT_5.0 T_) with two *b* values (*b* = 0 and 800 s/mm^2^), respectively. The signal-to-noise ratio (SNR) of different acquisition approaches were determined and statistically compared. The image quality was assessed and statistically compared with a 5-point scoring system.

**Results:**

The SNRs of any 5.0 T DWI images were significantly higher than those of any 3.0 T DWI images for same anatomic locations. Moreover, 5.0 T rFOV-DWIs had the significantly higher sharpness scores than 3.0 T rFOV-DWIs. Similar distortion scores were observed at both 3.0 T and 5.0 T. Finally, RT_5.0 T_ displayed the best overall image quality followed by NT_5.0 T_, FB_5.0 T_, RT_3.0 T_, NT_3.0 T_ and FB_3.0 T_ (RT_5.0 T_ = 3.9 ± 0.3, NT_5.0 T_ = 3.8 ± 0.3, FB_5.0 T_ = 3.4 ± 0.3, RT_3.0 T_ = 3.2 ± 0.4, NT_3.0 T_ = 3.1 ± 0.4, and FB_3.0 T_ = 2.7 ± 0.4, *p* < 0.001).

**Conclusion:**

The 5.0 T rFOV-DWI showed better overall image quality and improved SNR compared to 3.0 T rFOV-DWI, which holds clinical potential for identifying the abdominal abnormalities in routine practice.

**Critical relevance statement:**

This study provided evidence that abdominal 5.0 Tesla reduced field of view diffusion-weighted imaging (5.0 T rFOV-DWI) exhibited enhanced image quality and higher SNR compared to its 3.0 Tesla counterparts, holding clinical promise for accurately visualizing abdominal abnormalities.

**Key points:**

• rFOV-DWI was firstly integrated with high-field-MRI for visualizing various abdominal organs.

• This study indicated the feasibility of abdominal 5.0 T-rFOV-DWI.

• Better image quality was identified for 5.0 T rFOV-DWI.

**Graphical Abstract:**

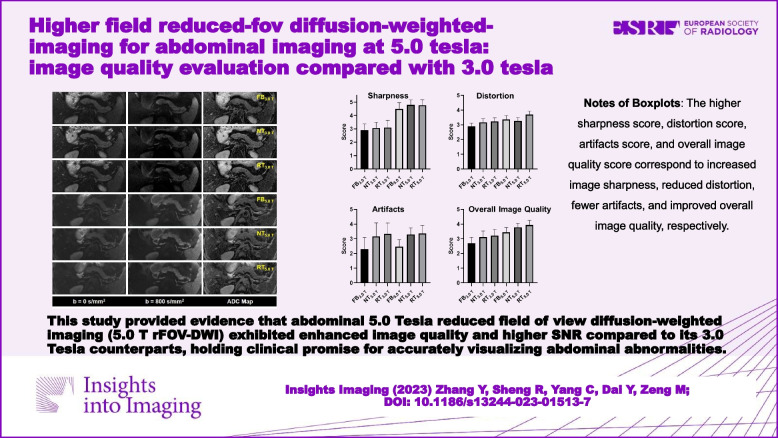

## Introduction

Diffusion-weighted imaging (DWI) is of great significance in medical application [1–3]. Nevertheless, spin-echo echo-planner imaging (SE-EPI)-based DWI is challenged by the following aspects: (1) SE-EPI-based DWI is prone to the image blurring, distortions, and signal voids [4]; (2) the insufficient signal-to-noise ratio (SNR) (especially in high *b* values) may result in the confusion of the diagnostic conclusions; (3) it is difficult to access high-resolution DWI together with satisfactory SNR and acceptable acquisition time; (4) the SNR and resolution related issues may influence the quantitative accuracy of DWI-derived metrics. The advance in high field MRI is advantageous for addressing above issues.

Tremendous efforts have been devoted to developing the high field MRI including the 4.7 T, 7.0 T, 9.4 T, 11.0 T, 14.7 T, and 17.6 T MRI in the past few decades [5–10]. Remarkable SNR benefits ease the balance among the acquisition time, resolution, and SNR. However, except 7.0 T MRI, most of the high field MRIs are confined to the pre-clinical animal studies. Regretfully, 7.0 T MRI, the limited choice of high field MRI for human imaging, is currently mainly restricted to the head and extremities. The technical challenges containing the severe field inhomogeneity, high specific absorption ratio (SAR), rapid T2 relaxation decay, and long T1 relaxation recovery impede the extensive applications of high field MRI. Moreover, it has been broadly reported that susceptibility artifact, distortion, blurring, and signal voids suffered by SE-EPI-based DWI scale with the main field strength (B0 field strength) [11]. Therefore, abdominal high field DWI, coveted by plenty of researchers, have hardly been carried out for human imaging.

Reduced field-of-view (rFOV) technique is a feasible approach to counter the constraints of conventional DWI. Utilizing a spatially selective localized radiofrequency (RF) excitation pulses or/and an outer-volume suppression (OVS), the sampling density of K-Space is reduced, which results in a smaller dataset size for given resolution. Therefore, the acquisition time, motion artifacts, susceptibility effects, and distortion will be diminished [12, 13]. Furthermore, owning to the spatially selective RF pulses and reduced K-Space encoding, the RF power deposition will be also improved. Previous findings unveiled that the combination of rFOV and high field DWI allows ultra-high-resolution imaging for human brain [14, 15].

Recently, the development of a 5.0 T whole-body MRI system is a notable advancement. According to physical theory of MRI, although the main field strength of 5.0 T system is lower compared to 7.0 T system, which leads to a decrease in SNR gain, there is an improvement in addressing issues related to field inhomogeneity, high SAR, and relaxation time. Previous research findings further support the value of 5.0 T MRI in imaging the brain, liver, kidney, and pancreas [16, 17]. We hypothesized that the 5.0 T MRI may serve as a potential option for high field rFOV-DWI. Therefore, this research aims to evaluate the image quality of 5.0 T rFOV-DWI with the 3.0 T rFOV-DWI as the reference. To the best of our knowledge, hardly has the high field abdominal rFOV-DWI been performed.

## Materials and methods

### Subjects

This prospective study was approved by the local ethical institution and the written informed consents from all included subjects were obtained. In total, 15 healthy volunteers (female: 4, male: 11; age: 37.8 ± 8.9 years, min: 18 years, max: 51 years; weight: 67.7 ± 9.2 kg, min: 55 kg, max: 85 kg; body mass index (BMI): 23.9 ± 2.6 kg/m^2^, min: 19.4 kg/m^2^, max: 29.8 kg/m^2^) were included into this study from November 2021 to December 2021.

### MRI examinations

All the subjects underwent the 3.0 T- and 5.0 T-MRI examinations. In order to prevent the potential bias, the time interval between two examinations at 3.0 T and 5.0 T was less than 4 days (2 ± 1.9 days). The 5.0 T MRI examinations were performed with a prototype whole-body MRI scanner (uMR Jupiter, United Imaging Healthcare). Free-breathing (FB), respiratory-triggered (RT), and navigator-triggered (NT) spin-echo echo-planner imaging (SE-EPI)-based three imaging protocols were performed subsequently. Except the difference in the strategies of countering the breathing motion, three sequences (5.0 T-FB-DWI (FB_5.0 T_), 5.0 T-RT-DWI (RT_5.0 T_), and 5.0 T-NT-DWI (NT_5.0 T_)) were configured to as the same imaging parameters as possible. The detailed imaging protocols were as follows: repetition time (TR): ~ 4500 ms (influenced by the respiratory cycle), echo time (TE): 50.5 ms, flip angle (FA): 90°, field of view (FOV): 120 × 280 mm^2^, matrix: 96 × 224, slice thickness: 6 mm, interlayer spacing: 20%, fat suppression: spectral adiabatic inversion-recovery (SPAIR) fat suppression, *b* values: 0 s/mm^2^ (two averages) and 800 s/mm^2^ (8 averages).

The 3.0 T MRI examinations were performed with a commercial MRI scanner (uMR 790, United Imaging Healthcare, Shanghai, China). Similar to 5.0 T examinations, FB, NT, and RT SE-EPI-based three DWI acquisitions were performed subsequently. Except the difference in the strategies of countering the breathing motion, three sequences (3.0 T-FB-DWI (FB_3.0 T_), 3.0 T-RT-DWI (RT_3.0 T_), and 3.0 T-NT-DWI (NT_3.0 T_)) were also configured to as the same imaging parameters as possible. The detailed imaging protocols were as follows: TR: ~ 4000 ms (influenced by the respiratory cycle), TE: 52.5 ms, FA: 90°, FOV: 120 × 280 mm^2^, matrix: 96 × 224, slice thickness: 6 mm, interlayer spacing: 20%, fat suppression: SPAIR fat suppression, *b* values: 0 s/mm^2^ (two averages) and 800 s/mm^2^ (8 averages)). The acquisition time for FB_3.0 T_ and FB_5.0 T_ were 123.0 s and 97.0 s, respectively. The acquisition time of the other four sequences were not consistent and associated with the respiratory cycle of the subjects.

For 3.0 T and 5.0 T examinations, the inline reconstructed apparent diffusion coefficients (ADC) maps were obtained by means of the workstation (United Imaging Healthcare) according to a mono-exponential diffusion model. All six DWI examinations at 3.0 T and 5.0 T were based on the same rFOV strategy termed as MicroView technique, which is able to reduce the FOV in phase encoding direction and achieve the outer volume suppression (OVS).

### Image analysis

Two experienced abdominal radiologists with 10 years’ and 6 years’ experiences were invited to perform the image analysis. Two observers were blinded to both the MRI protocols and subject’s characteristics during image analysis.

#### SNR quantification

The SNR of DWI images (*b* = 0 s/mm^2^ and 800 s/mm^2^) in upper abdominal organs including liver, pancreas, spleen, and kidney collected through six sequences (FB_5.0 T_, NT_5.0 T_, RT_5.0 T_, FB_3.0 T_, NT_3.0 T_, RT_3.0 T_) were independently measured by two observers according to the following formula: $SNR= {SI}_{tissue} / {SD}_{noise}$, where SI and SD were the abbreviations of signal intensity and standard deviation, respectively. In detail, 50-pixel circle region of interests (ROI) were drawn in each anatomic locations to measure the ${SI}_{tissue}$; ${SD}_{noise}$ was measured by localizing the ROI in uniform background. Vessels and artifacts were carefully avoided during the ROI delineation. Moreover, for each anatomic position of each subject, six imaging sequences (FB_5.0 T_, NT_5.0 T_, RT_5.0 T_, FB_3.0 T_, NT_3.0 T_, RT_3.0 T_) were simultaneously reviewed and the ROIs defined in different images should be at as same position as possible.

#### Image quality (IQ) evaluation

Each observer was asked to evaluate the image quality for three times. The image quality of each DWI acquisition was evaluated based on the overall quality of DWI images (*b* = 0 and 800 s/mm^2^) and ADC maps in terms of sharpness, distortion, and artifacts. Specifically, the sharpness, distortion, and artifact were scored based on the 5-point scaling criteria: sharpness: 5 = excellent, 4 = good, 3 = fair, 2 = poor, 1 = very poor and non-diagnostic; distortion: 5 = no distortion, 4 = slight distortion, 3 = medium distortion, 2 = severe distortion, and 1 = very severe distortion and non-diagnostic; artifacts: 5 = no artifacts, 4 = slight artifacts, 3 = moderate artifacts, 2 = severe artifacts, and 1 = very severe artifacts and non-diagnostic, it should be noted that the artifacts were scored based on the presence of all kinds of artifacts including susceptibility artifacts, motion artifacts, ghosts, and so on. Overall IQ was determined by means of averaging the scores of sharpness, distortion, and artifacts.

### Statistical analysis

The Shapiro–Wilk test was firstly to test the data normality. The Friedman test was applied to assess whether there existed the significant differences among the six imaging acquisitions in terms of SNR, sharpness score, distortion score, artifact score, and overall IQ. Then, the post hoc multiple comparisons were conducted via Friedman’s two-way ANOVA (by ranks). The intra-class coefficients (ICCs) were calculated to quantify both intra-observer and inter-observer agreement. The intra-observer and inter-observer agreements were determined as excellent for ICCs = 0.8–1.0, substantial for ICCs = 0.6–0.8, moderate for ICCs = 0.4–0.6, fair for ICCs = 0.2–0.4, and poor for ICCs = 0.0–0.2. Two-sided *p* values of less than 0.05 indicate significant differences. All the statistical analysis were carried out with SPSS version 26.0 (SPSS Inc., Chicago IL, USA).

## Results

**Fig. 1:**
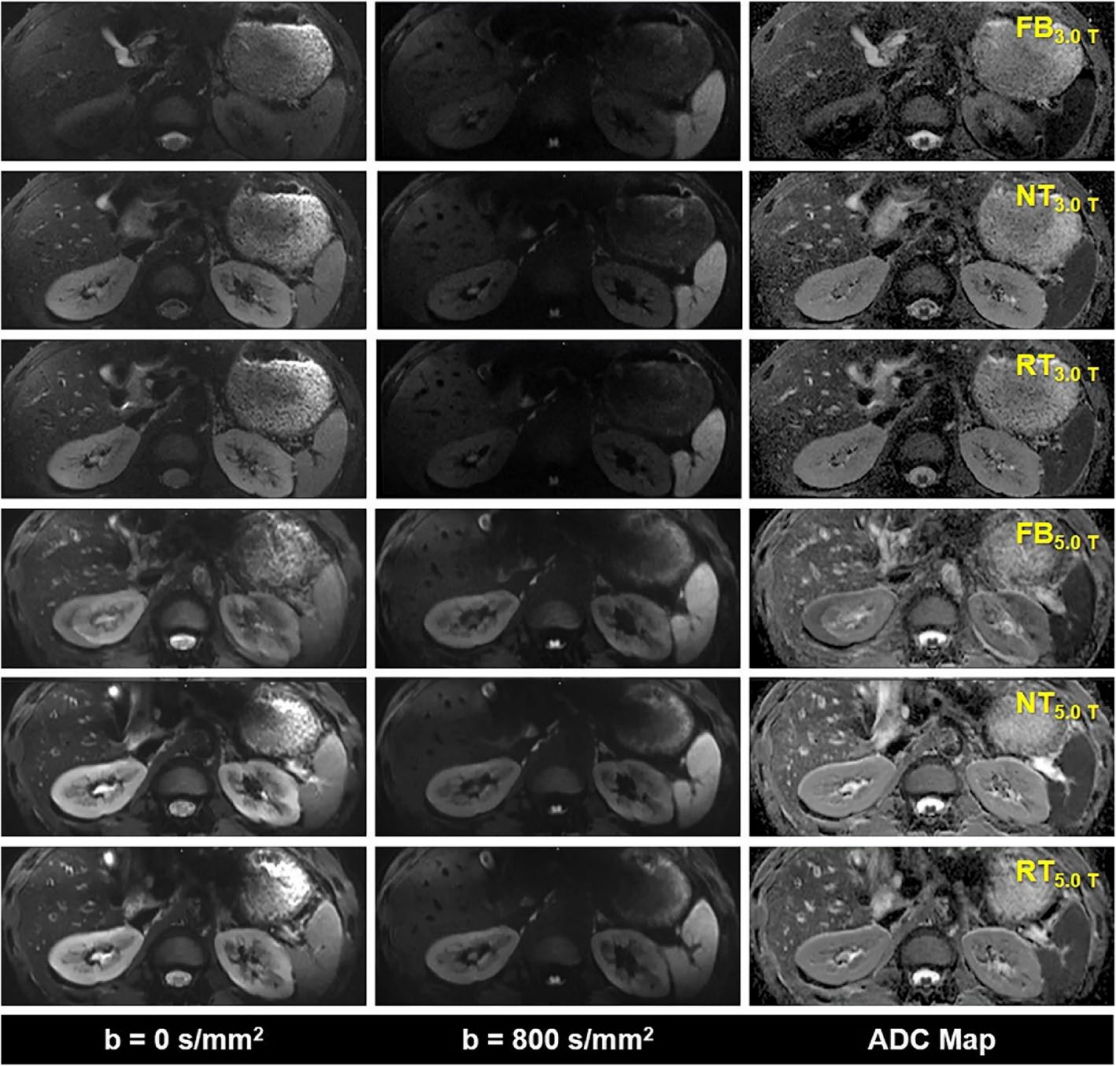
Representative MR images of a 25-year-old man weighing 65.0 kg and with a BMI of 20.5. The DWI images obtained by six acquisition approaches are listed in different rows. The DWI images of *b* = 0 s/mm^2^, *b* = 800 s/mm^2^, and ADC parametric maps are displayed in different columns

**Fig. 2:**
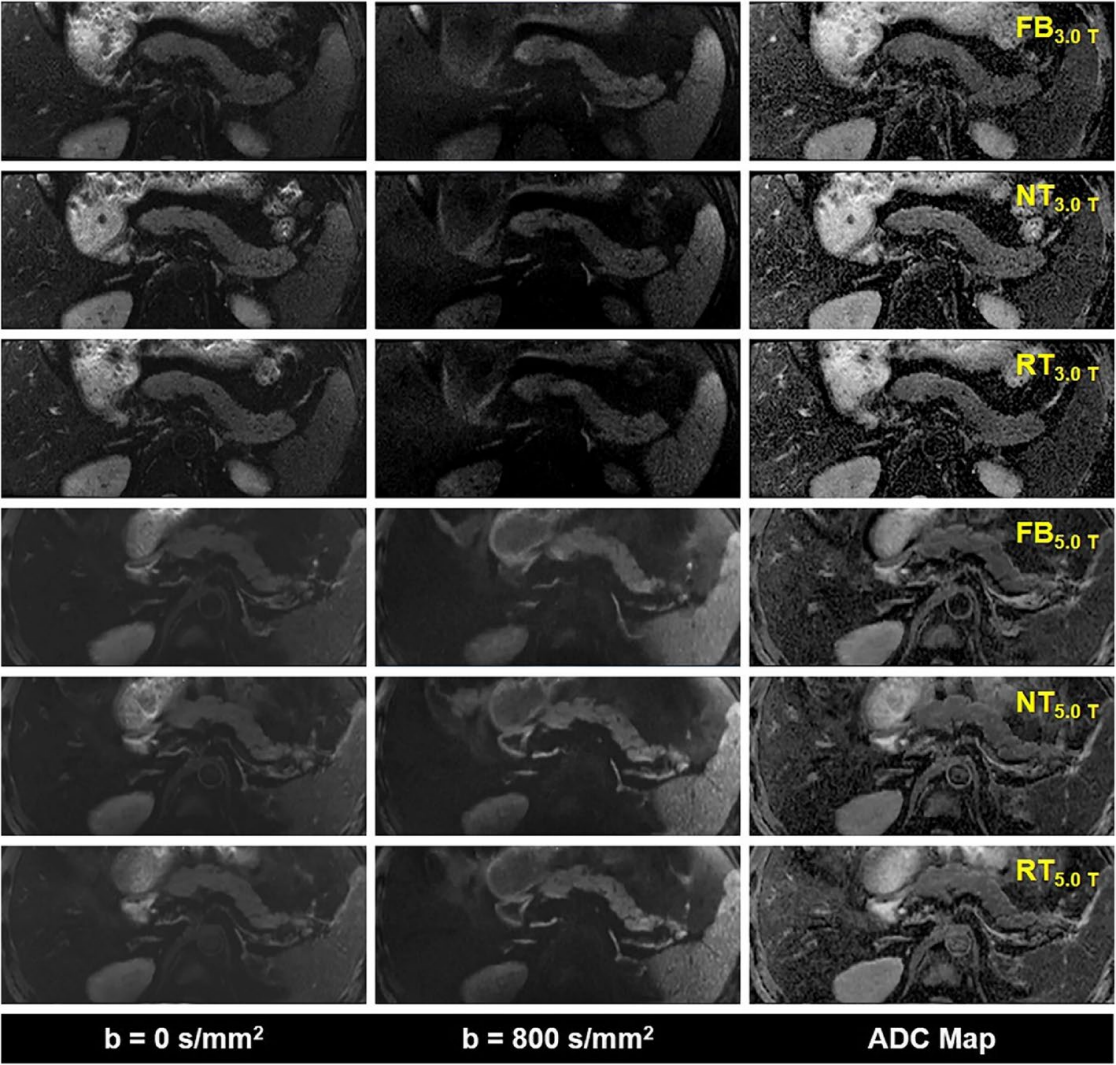
Representative MR images of a 36-year-old man weighing 75.4 kg and with a BMI of 24.9. The DWI images obtained by six acquisition approaches are listed in different rows. The DWI images of *b* = 0 s/mm^2^, *b* = 800 s/mm^2^, and ADC parametric maps are displayed in different columns

**Fig. 3:**
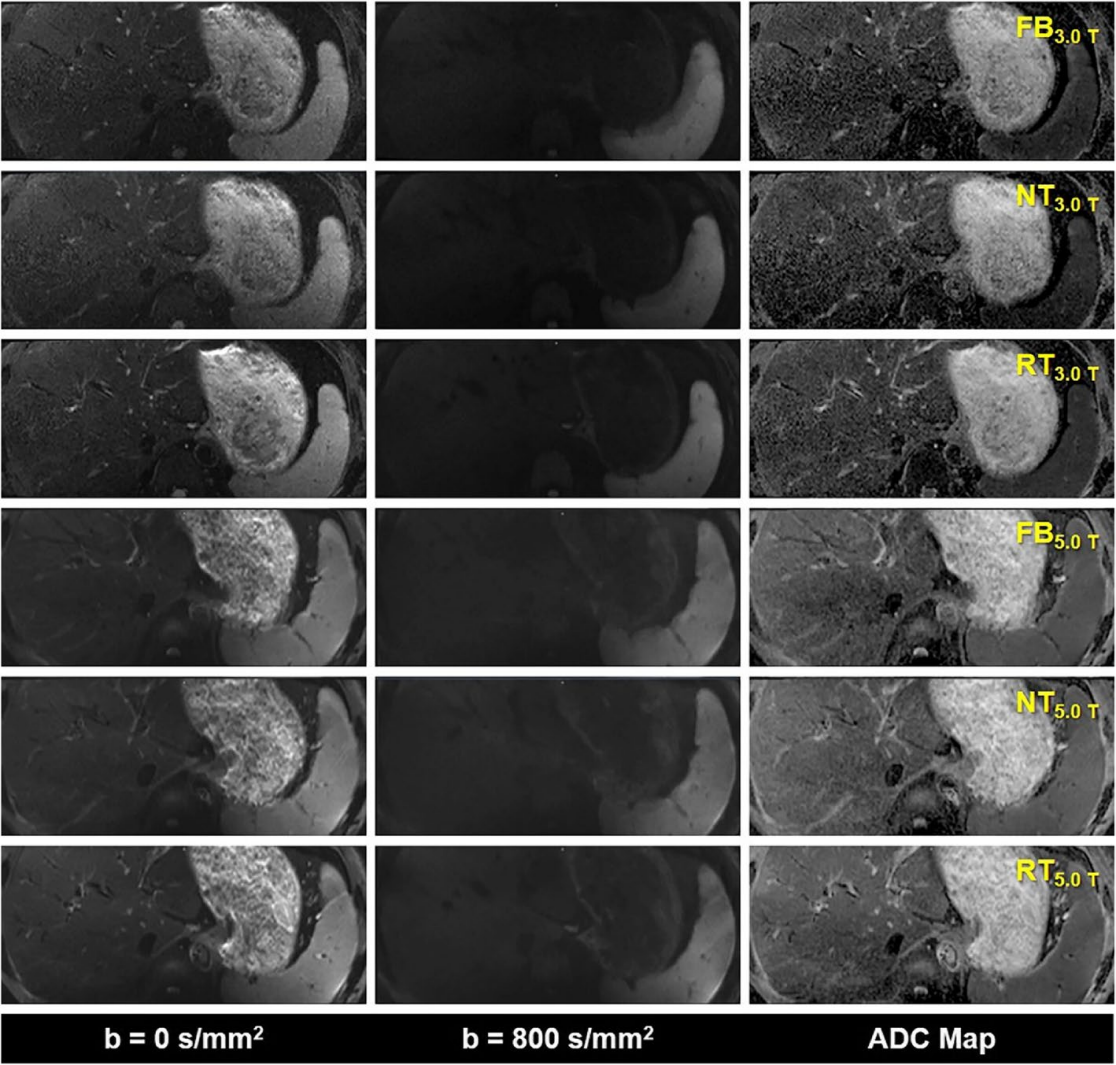
Representative MR images of an 18-year-old man weighing 74.2 kg and with a BMI of 25.4. The DWI images obtained by six acquisition approaches are listed in different rows. The DWI images of *b* = 0 s/mm^2^, *b* = 800 s/mm^2^, and ADC parametric maps are displayed in different columns

**Fig. 4:**
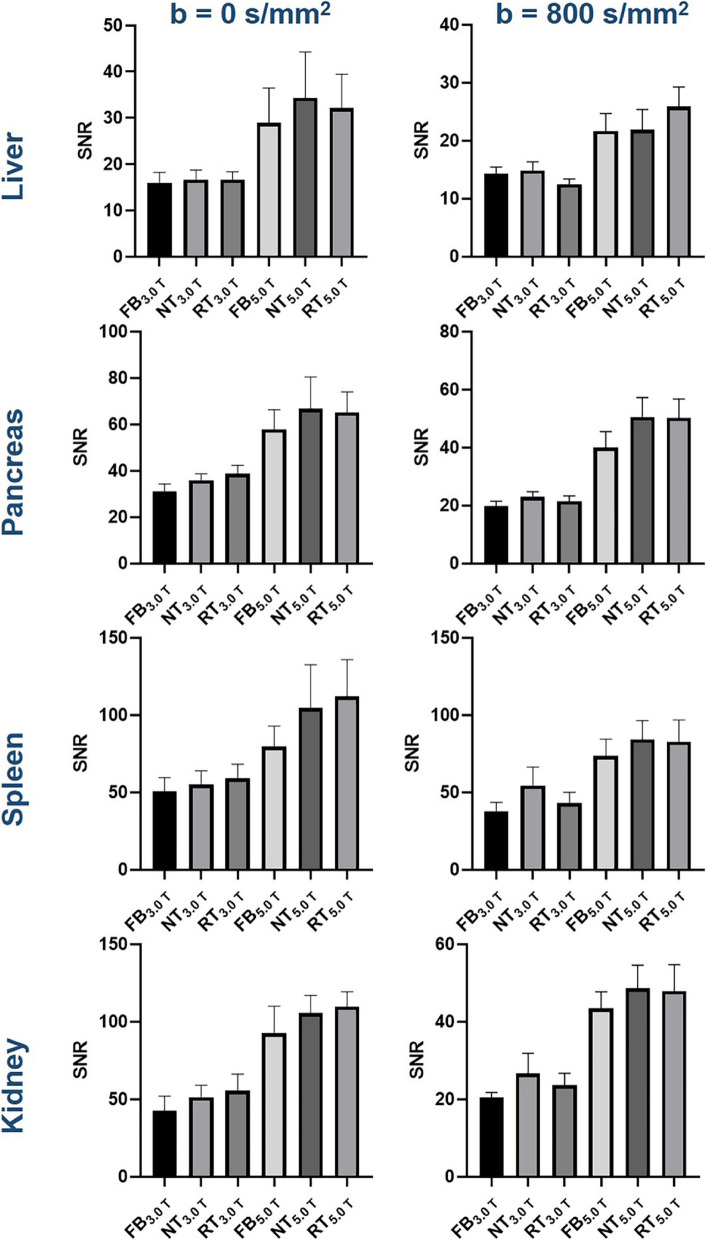
Box-plots show the SNRs of different anatomical structures in DWI images obtained with six acquisition approaches

**Fig. 5:**
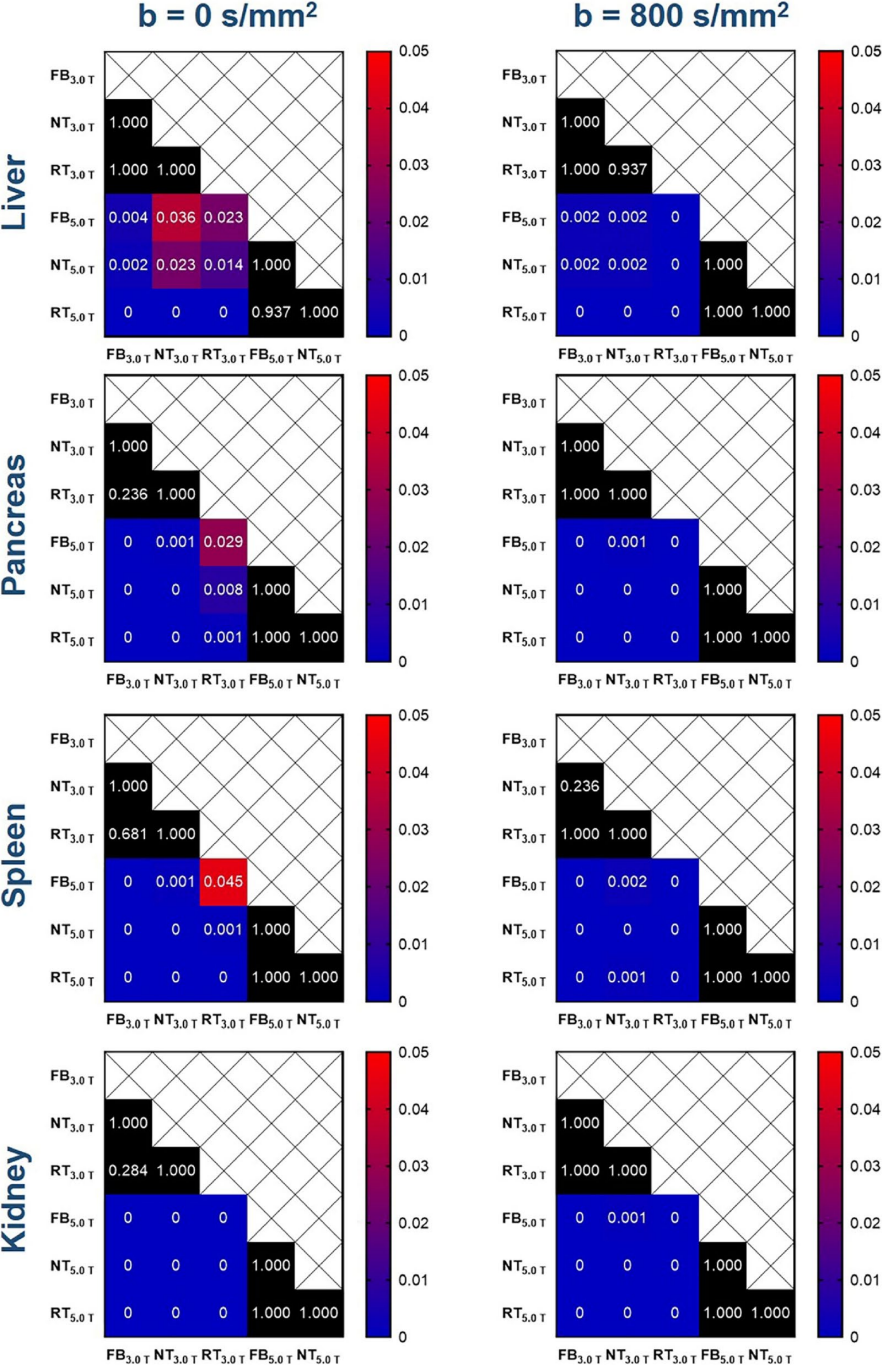
Heat maps show the post hoc multiple comparisons of SNRs of different anatomical structures in DWI images (*b* = 0 s/mm^2^ and 800 s/mm^2^) obtained with six acquisition approaches. The values labeled in each cell represent the *p* values of post hoc comparison with Bonferroni adjustment. The scale bars indicate the *p* values (0.00–0.05); the black filled cells indicate no significant difference. The values of 0 suggest the corresponding *p* values are less than 0.001 according to the SPSS software

**Fig. 6:**
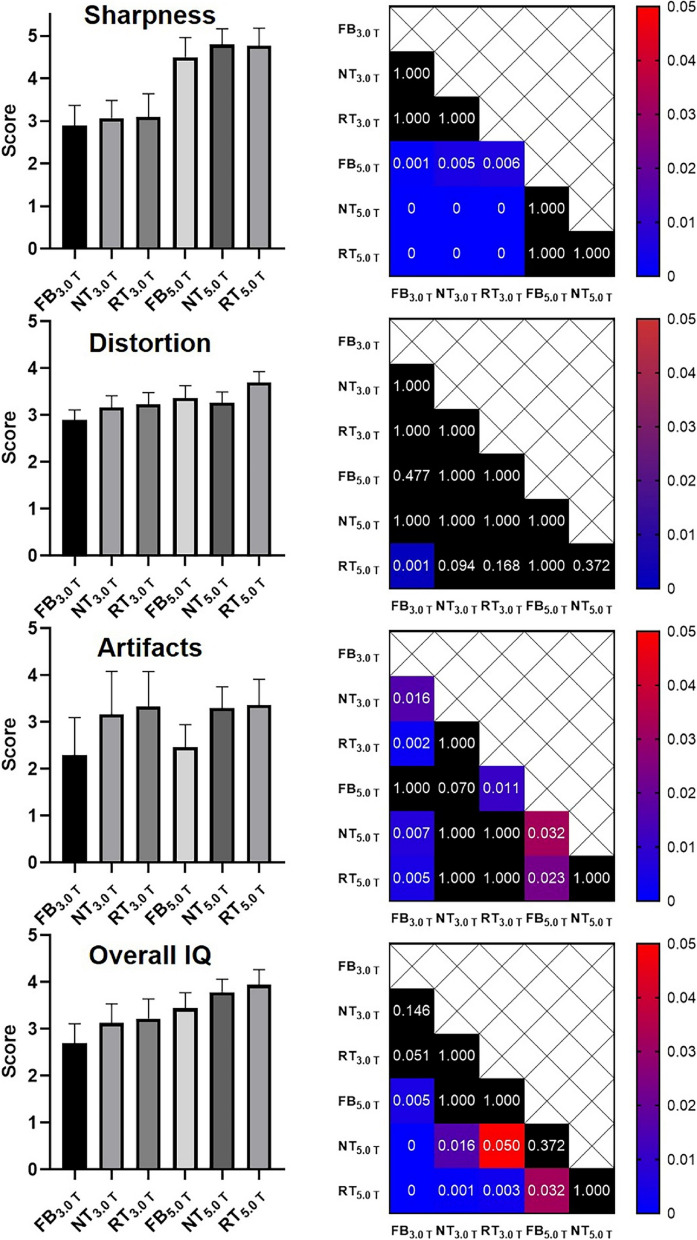
Image quality evaluation of six DWI acquisition strategies. The heat maps displayed in the right of box plots show the corresponding post hoc comparison results. For example, the heat map exhibited in the first row demonstrates the post hoc multiple comparison results in terms of sharpness scores. The values labeled in each cell represent the *p* values of post hoc comparison with Bonferroni adjustment. The scale bars indicate the *p* values (0.00–0.05), the black filled cells indicate no significant difference. The values of 0 suggest the corresponding *p* values are less than 0.001 according to the SPSS software

The representative DWI images were displayed in Figs. 1, 2, and 3. Qualitatively, 5.0 T rFOV-DWIs (FB_5.0 T,_ NT_5.0 T,_ RT_5.0 T_) yielded the better image quality as well as the visibility of abdominal organs and structures. For instance, the cortex and medulla in kidney were more clearly visualized by 5.0 T rFOV-DWIs with regard to the 3.0 T rFOV-DWIs (Fig. 1). Additionally, the overall structure of pancreas in DWIs images at 5.0 T were much clearer than those at 3.0 T (Fig. 2). Besides, much more image noise in FB_3.0 T,_ NT_3.0 T_ and RT_3.0 T_ were observed than their counterparts at 5.0 T (Figs. 1, 2, and 3). Furthermore, the difference in image quality at two field strengths was more obvious at high *b* values (*b* = 800 s/mm^2^) (Figs. 1, 2, and 3). For four upper abdominal organs (liver, pancreas, spleen, and kidney) in DWI images of *b* = 0 s/mm^2^ and *b* = 800 s/mm^2^, there existed the significant differences among the SNRs of six DWI examinations (*p* < 0.05) (Table 1, Fig. 4). The SNRs of liver were relatively low compared to those of pancreas, spleen, and kidney. The post hoc multiple comparisons suggested that the SNRs of any 5.0 T DWI images were significantly higher than those of any 3.0 T DWI images for same anatomic location in DWI images of *b* = 0 s/mm^2^ and 800 s/mm^2^ (Fig. 5). Furthermore, no significant differences were observed among the SNRs of different imaging strategies at the same strength (*p* > 0.05). The results regarding the intra-observer and inter-observer agreements between the image quality evaluation in terms of sharpness, distortion, artifacts, and overall IQ were exhibited in Tables 2 and 3. The findings are as follows: ICC ranged from 0.615 to 1.000, signified that the interobserver agreements were determined as substantial to excellent for evaluating the image quality of six imaging protocols. As shown in Fig. 6 and Table 4, NT_5.0 T_ and RT_5.0 T_ had the higher sharpness scores followed by FB_5.0 T_, RT_3.0 T_, NT_3.0 T_, and FB_3.0 T_ (NT_5.0 T_ = 4.8 ± 0.4, RT_5.0 T_ = 4.8 ± 0.4, FB_5.0 T_ = 4.5 ± 0.5, RT_3.0 T_ = 3.1 ± 0.5, NT_3.0 T_ = 3.1 ± 0.4 and FB_3.0 T_ = 2.9 ± 0.5, *p* < 0.001). The post hoc multiple comparisons showed that 5.0 T DWI examinations yielded the significantly higher sharpness scores than their counterparts at 3.0 T. With respect to geometric distortion, six imaging protocols have the similar distortion scores (min: 2.9 ± 0.4, max: 3.7 ± 0.4). Only the significant difference between the distortion scores of FB_3.0 T_ and RT_5.0 T_ was identified (*p* = 0.001). As for artifacts, six image protocols were scored based on the presence of motion artifacts, susceptibility artifacts, and ghosts. Specifically, the severest artifacts were observed in FB_3.0 T_ and FB_5.0 T_ followed by NT_3.0 T_, NT_5.0 T_, RT_3.0 T_, and RT_5.0 T_ (FB_3.0 T_ = 2.3 ± 0.8, FB_5.0 T_ = 2.5 ± 0.5, NT_3.0 T_ = 3.2 ± 0.9, NT_5.0 T_ = 3.3 ± 0.5, RT_3.0 T_ = 3.3 ± 0.7 and RT_5.0 T_ = 3.4 ± 0.5, *p* < 0.001). Significant differences in artifacts scores existed among the free-breathing DWIs and non-free-breathing DWIs sequences (*p* < 0.05). Finally, the RT_5.0 T_ displayed the best overall IQ followed by NT_5.0 T_, FB_5.0 T_, RT_3.0 T_, NT_3.0 T_ and FB_3.0 T_ (RT_5.0 T_ = 3.9 ± 0.3, NT_5.0 T_ = 3.8 ± 0.3, FB_5.0 T_ = 3.4 ± 0.3, RT_3.0 T_ = 3.2 ± 0.4, NT_3.0 T_ = 3.1 ± 0.4, and FB_3.0 T_ = 2.7 ± 0.4, *p* < 0.001). There were significant differences between the FB_5.0 T_ and FB_3.0 T_ (*p* = 0.005), NT_5.0 T_ and FB_3.0 T_ (*p* < 0.001), RT_5.0 T_ and FB_3.0 T_ (*p* < 0.001), NT_5.0 T_ and NT_3.0 T_ (*p* = 0.016), RT_5.0 T_ and NT_3.0 T_ (*p* = 0.001), NT_5.0 T_ and RT_3.0 T_ (*p* = 0.050), and RT_5.0 T_ and RT_3.0 T_ (*p* = 0.003) as well as RT_5.0 T_ and RT_3.0 T_ (*p* = 0.032).

**Table 1:** SNRs of different anatomical structures in DWI images obtained from six acquisition approaches

	**FB**_**3.0 T**_	**NT**_**3.0 T**_	**RT**_**3.0 T**_	**FB**_**5.0 T**_	**NT**_**5.0 T**_	**RT**_**5.0 T**_	***p***
**Liver**_**b0**_	16.0 ± 6.1	16.7 ± 5.6	16.6 ± 4.6	29.0 ± 20.1	34.4 ± 26.5	32.1 ± 19.5	***
**Liver**_**b800**_	14.3 ± 3.1	14.9 ± 3.9	12.5 ± 2.5	21.7 ± 8.1	21.9 ± 9.4	25.9 ± 9.0	***
**Pancreas**_**b0**_	30.9 ± 8.9	35.8 ± 7.7	38.7 ± 9.8	57.8 ± 22.7	66.6 ± 37.0	65.1 ± 23.9	***
**Pancreas**_**b800**_	19.7 ± 4.7	23.0 ± 4.8	21.4 ± 5.0	40.0 ± 14.8	50.6 ± 17.8	50.3 ± 17.3	***
**Spleen**_**b0**_	50.6 ± 24.0	55.1 ± 23.6	59.0 ± 24.6	79.5 ± 36.2	104.4 ± 75.5	112.0 ± 63.9	***
**Spleen**_**b800**_	37.6 ± 16.1	54.2 ± 32.5	42.9 ± 19.1	73.4 ± 29.6	84.2 ± 32.6	82.6 ± 38.0	***
**Kidney**_**b0**_	45.6 ± 16.2	53.8 ± 17.1	60.0 ± 21.0	99.1 ± 20.4	105.6 ± 26.5	104.8 ± 29.5	***
**Kidney**_**b800**_	20.4 ± 3.6	26.6 ± 14.1	23.6 ± 8.4	43.4 ± 11.6	48.6 ± 15.9	47.9 ± 18.3	***

*FB*_*3.0 T*_, 3.0 T-free-breathing-DWI; *NT*_*3.0 T*_, 3.0 T-navigator-triggered-DWI; *RT*_*3.0 T*_, 3.0 T-respiratory-triggered-DWI; *FB*_*5.0 T*_, 5.0 T-free-breathing-DWI; *NT*_*5.0 T*_, 5.0 T-navigator-triggered-DWI; *RT*_*5.0 T*_, 5.0 T-respiratory-triggered-DWI

^***^ indicates the *p* value of less than 0.001

**Table 2:** Interobserver agreement of image quality assessment from two observers

		**FB**_**3.0 T**_	**NT**_**3.0 T**_	**RT**_**3.0 T**_	**FB**_**5.0 T**_	**NT**_**5.0 T**_	**RT**_**5.0 T**_
**Sharpness**	ICC	0.925	0.822	0.943	0.756	0.772	0.904
	Lower bound of 95% CI	0.776	0.470	0.830	0.272	0.321	0.714
	Upper bound of 95% CI	0.975	0.940	0.981	0.918	0.923	0.968
**Distortion**	ICC	0.889	0.918	0.750	0.928	0.822	0.750
	Lower bound of 95% CI	0.669	0.755	0.255	0.785	0.470	0.255
	Upper bound of 95% CI	0.963	0.972	0.916	0.976	0.940	0.916
**Artifact**	ICC	0.974	0.980	0.936	0.866	0.920	0.945
	Lower bound of 95% CI	0.922	0.941	0.810	0.601	0.760	0.836
	Upper bound of 95% CI	0.991	0.993	0.979	0.955	0.973	0.981
**Overall IQ**	ICC	0.966	0.932	0.955	0.911	0.836	0.955
	Lower bound of 95% CI	0.899	0.798	0.867	0.734	0.510	0.866
	Upper bound of 95% CI	0.989	0.977	0.985	0.970	0.945	0.985

*FB*_*3.0 T*_, 3.0 T-free-breathing-DWI; *NT*_*3.0 T*_, 3.0 T-navigator-triggered-DWI; *RT*_*3.0 T*_, 3.0 T-respiratory-triggered-DWI; *FB*_*5.0 T*_, 5.0 T-free-breathing-DWI; *NT*_*5.0 T*_, 5.0 T-navigator-triggered-DWI; *RT*_*5.0 T*_, 5.0 T-respiratory-triggered-DWI; *Overall IQ*, overall image quality; *CI*, confidence of interval

**Table 3:** Intraobserver agreement of image quality assessment from two observers

		**FB**_**3.0 T**_	**NT**_**3.0 T**_	**RT**_**3.0 T**_	**FB**_**5.0 T**_	**NT**_**5.0 T**_	**RT**_**5.0 T**_
**Sharpness (R1)**	ICC	0.917	1.000	0.937	0.917	0.803	0.894
	Lower bound of 95% CI	0.802	1.000	0.850	0.802	0.532	0.748
	Upper bound of 95% CI	0.970	1.000	0.977	0.970	0.928	0.961
**Distortion (R1)**	ICC	0.773	0.917	0.894	0.914	0.870	0.870
	Lower bound of 95% CI	0.460	0.802	0.748	0.795	0.692	0.692
	Upper bound of 95% CI	0.917	0.970	0.961	0.969	0.953	0.953
**Artifact (R1)**	ICC	0.967	0.946	0.967	0.917	0.907	0.914
	Lower bound of 95% CI	0.921	0.872	0.921	0.802	0.778	0.795
	Upper bound of 95% CI	0.988	0.980	0.988	0.970	0.966	0.969
**Overall IQ (R1)**	ICC	0.948	0.962	0.958	0.907	0.887	0.924
	Lower bound of 95% CI	0.877	0.910	0.901	0.779	0.731	0.820
	Upper bound of 95% CI	0.981	0.986	0.985	0.966	0.959	0.972
	ICC	0.894	0.667	1.000	0.845	0.821	0.870
**Sharpness (R2)**	Lower bound of 95% CI	0.748	0.209	1.000	0.633	0.574	0.692
	Upper bound of 95% CI	0.961	0.879	1.000	0.944	0.935	0.953
	ICC	0.615	0.870	0.929	1.000	0.907	1.000
**Distortion (R2)**	Lower bound of 95% CI	0.087	0.692	0.832	1.000	0.778	1.000
	Upper bound of 95% CI	0.860	0.953	0.974	1.000	0.966	1.000
	ICC	0.965	1.000	0.921	0.914	1.000	0.942
**Artifact (R2)**	Lower bound of 95% CI	0.917	1.000	0.813	0.795	1.000	0.861
	Upper bound of 95% CI	0.987	1.000	0.971	0.969	1.000	0.979
	ICC	0.965	0.936	0.958	0.932	0.940	0.961
**Overall IQ (R2)**	Lower bound of 95% CI	0.916	0.847	0.900	0.839	0.859	0.908
	Upper bound of 95% CI	0.987	0.977	0.985	0.975	0.978	0.986

*FB*_*3.0 T*_, 3.0 T-free-breathing-DWI; *NT*_*3.0 T*_, 3.0 T-navigator-triggered-DWI; *RT*_*3.0 T*_, 3.0 T-respiratory-triggered-DWI; *FB*_*5.0 T*_, 5.0 T-free-breathing-DWI; *NT*_*5.0 T*_, 5.0 T-navigator-triggered-DWI; *RT*_*5.0 T*_, 5.0 T-respiratory-triggered-DWI; *Overall IQ*, overall image quality; *CI*, confidence of interval; *R1*, reader 1; *R2*, reader 2

**Table 4:** Image quality of six DWI acquisition approaches in terms of sharpness, distortion, artifact, and overall IQ

	**FB**_**3.0 T**_	**NT**_**3.0 T**_	**RT**_**3.0 T**_	**FB**_**5.0 T**_	**NT**_**5.0 T**_	**RT**_**5.0 T**_	***p* values**
**Sharpness**	2.9 ± 0.5	3.1 ± 0.4	3.1 ± 0.5	4.5 ± 0.5	4.8 ± 0.4	4.8 ± 0.4	< 0.001
**Distortion**	2.9 ± 0.4	3.2 ± 0.4	3.2 ± 0.5	3.4 ± 0.5	3.3 ± 0.4	3.7 ± 0.4	< 0.001
**Artifact**	2.3 ± 0.8	3.2 ± 0.9	3.3 ± 0.7	2.5 ± 0.5	3.3 ± 0.5	3.4 ± 0.5	< 0.001
**Overall IQ**	2.7 ± 0.4	3.1 ± 0.4	3.2 ± 0.4	3.4 ± 0.3	3.8 ± 0.3	3.9 ± 0.3	< 0.001

*FB*_*3.0 T*_, 3.0 T-free-breathing-DWI; *NT*_*3.0 T*_, 3.0 T-navigator-triggered-DWI; *RT*_*3.0 T*_, 3.0 T-respiratory-triggered-DWI; *FB*_*5.0 T*_, 5.0 T-free-breathing-DWI; *NT*_*5.0 T*_, 5.0 T-navigator-triggered-DWI; *RT*_*5.0 T*_, 5.0 T-respiratory-triggered-DWI; *Overall IQ*, overall image quality

## Discussion

As the restricted option for high field MRI applied in human imaging, 7.0 T DWI is mainly limited to the brain applications because of the following technical challenges: (1) for high field MRI, the “standing wave” effect will emerge in case of that wavelength of RF excitation approaches the size of tissue to be imaged, which further results in an unexpected strong inhomogeneity in either reception (B1^−^) and transmission (B1^+^) field as well as regional SAR peaks. The image quality in terms of regional SNR, contrast, and uniformity will hence be severely compromised [18, 19]. (2) The RF power deposition is proportional to the square of B0 field strength [20]. (3) The decrease in T2 relaxation time scales with the B0 field strength, resulting in a much faster signal decay [21]. (4) As for conventional DWI, both the short minimum TE and fast readout cannot be easily accessed due to the technical constraints of gradient system. Unexpectedly, rapid decay of traverse relaxation and long recovery of longitudinal relaxation are the representative characteristics for high field MRI. The not short enough TE and fast signal decay cause the negative effects on the SNR (regional signal loss and voids). Moreover, the long readout duration leads to the mis-registration and geometric distortion as well as blurring because of the low imaging bandwidth in readout direction [22]. In addition, the pronounced susceptibility effects in high field MRI also account for the image distortion and artifacts.

Meaningfully, the development of rFOV-DWI technique is, to some extent, valuable for mitigating the aforementioned problems: (1) the decrease in phase encoding steps together with reduced FOV will achieve a shorter EPI echo train. Furthermore, the readout duration will be effectively decreased. Therefore, high-resolution imaging with less distortion and misregistration as well as without the increase in acquisition time (the results of reduced k-space dataset) will be enabled [23]. (2) By means of excluding the unnecessary imaging regions (such as air-tissue interfaces) from the shim volume, the susceptibility artifacts will also be diminished [24]. (3) rFOV-DWI can benefit the decrease in RF power deposition.

Based on the discussions above, due to the highly complementary characteristics of rFOV method and high field MRI, a good balance among the resolution, SNR, and acquisition time can be achieved with less distortions, artifacts, and blurring. Several attempts have been made to combine the rFOV method and high field DWI [14, 15]. To date, the high field rFOV-DWI, however, have hardly been applied in abdomen. One main cause may lie in that the B0 field strength of 7.0 T is “over high” for abdominal application in view of current level of technology: the negative effects raised by over high field strength cannot be easily surpassed. Recently, the 5.0 T whole-body MRI scanner was developed, providing another choice for high field abdominal DWI.

In this study, the results involving the SNR comparison showed that the increase in B0 field strength provided a remarkable SNR gain. For four upper abdomen organs containing the liver, pancreas, spleen, and kidney, the SNRs of both b0 and b800 DWI images at 5.0 T were significantly higher than those at 3.0 T. The SNR gain (SNR_5.0 T_/ SNR_3.0 T_) was determined as ranging from 1.26 to 2.35 for same acquisition strategies (FB, NT or RT) in four upper abdominal organs. The potential causes for the variation of SNR gain are as follows: all other factors being equal, the SNR improvement brought from the high B0 field is as follows [15]:

$$\frac{{SNR}_{5.0 T}}{{SNR}_{3.0 T}}= \frac{5}{3} \mathrm{exp}(\frac{{TE}_{ 3.0 T}}{{T}_{2, 3.0 T}}-\frac{{TE}_{ 5.0 T}}{{T}_{2, 5.0 T}})$$

The T2 shortening in 5.0 T MRI caused a fast signal decay and thus acted as the negative impacts on SNR gain. Furthermore, the T2 relaxation time of various biological tissue in abdomen remains unclear at 5.0 T. We carefully hypothesized that the T2 shortening effects varied for different organs. Besides, the minimum TEs in 3.0 T and 5.0 T imaging protocols were inconsistent in this study because of the differences in hardware and software of MRI scanners. Besides, although cares were taken to define the paired ROIs at as the same position as possible, the completely paired ROIs in images from different acquisitions were inaccessible due to the breathing motion, which may lead to some bias. Above results corresponded to the previous findings: the SNR at 3.0 T MRI is not absolute 2-folds of that at 1.5 T MRI as well as the SNR at 7.0 T is not absolute ${}^{7}\!\left/ \!{}_{3}\right.$-folds of that at 3.0 T MRI for the same tissue [25–27]. Another finding was that no significant differences were observed between the SNRs of three acquisition approaches including FB, NT, and RT at the same field strength. It has been reported that irregular breathing motions will result in the intra-voxel dephasing related signal decay and the regular breathing motion may not bring the significant difference [28]. In this study, all the included subjects were healthy volunteers and underwent respiratory training before the MRI examination.

Increased sharpness score was observed for FB_5.0 T_, NT_5.0 T_, and RT_5.0 T_ compared to other sequences. Above results can be explained by the following points: (1) the configuration of same FOV and scanning matrix ensured the consistent resolution for six sequences. (2) In the case of the same resolution, the sharpness scores will be mainly determined by the SNR. Based on the aforementioned discussions, 5.0 T MRI gave a remarkable increase in SNR. Additionally, all six sequences had the close distortion scores (distortion scores = 2.9–3.7). The similar distortion scores were mainly due to the fact that all six sequences were based on the rFOV method able to mitigate the distortion appearing in conventional DWI by means of a shorter EPI echo train and readout duration. Similarly, a large number of previous investigations have revealed the efficacy of rFOV method in reducing the distortion [29–32]. Only significant difference between the RT_5.0 T_ and FB_3.0 T_ was identified, which may be caused by the relatively low SNR and breathing motion. Artifacts should be viewed as another critical factor for the image quality evaluation; our results suggested that the breathing motion artifacts serve as the main contributor to the differences of artifacts scores among six imaging strategies. The FB_3.0 T_ and FB_5.0 T_ showed significantly severe artifacts than other sequences, which suggested that the NT or the RT technique should be adopted to counter the breathing motion artifacts for both 3.0 T and 5.0 T rFOV-DWI during the clinical application. Finally, the overall IQs of six sequences were quantified based on the sharpness, distortion, and artifacts. Our results showed that the RT_5.0 T_ yielded the best image quality score followed by NT_5.0 T_, FB_5.0 T_, RT_3.0 T_, NT_3.0 T_, and FB_3.0 T_. Besides, no significant difference was observed between the IQ of RT_5.0 T_ and NT_5.0 T_, demonstrating that the RT_5.0 T_ and NT_5.0 T_ were recommended for abdominal DWI imaging.

Several limitations of this study should be acknowledged. (1) The sample size of this prospective study was relatively small, which holds risk for statistical bias and only reflects our initial experience. (2) The healthy volunteers were included into this study; the performance of visualizing the representative abdominal abnormalities should be systematically assessed in the following study. (3) Only two *b* values (*b* = 0 and 800 s/mm^2^) were configured; the efficacy of 5.0 T multiple *b* (especially high *b* values of more than 2000 s/mm^2^) DWI should be evaluated in the following study.

## Conclusion

In view of the results that 5.0 T rFOV-DWI showed an improved image quality for upper abdomen compared to 3.0 T rFOV-DWI, 5.0 T rFOV-DWI holds clinical potential for visualizing the abdominal abnormalities with high resolution and SNR.

## Data Availability

The data can be accessed from the corresponding authors under reasonable request.

## Abbreviations

ADC: Apparent diffusion coefficients
DWI: Diffusion-weighted imaging
FB: Free-breathing
FB_3.0 T_: 3.0 T-free-breathing-DWI
FB_5.0 T_: 5.0 T-free-breathing-DWI
ICC: Intra-class coefficients
IQ: Image quality
NT: Navigator-triggered
NT_3.0 T_: 3.0 T-navigator-triggered-DWI
NT_5.0 T_: 5.0 T-navigator-triggered-DWI
rFOV: Reduced field of view
RT: Respiratory-triggered
RT_3.0 T_: 3.0 T-respiratory-triggered-DWI
RT_5.0 T_: 5.0 T-respiratory-triggered-DWI
SAR: Specific absorption ratio
SE-EPI: Spin-echo echo-planner imaging
SNR: Signal-to-noise ratio
